# The Comparative Neurology of Neocortical Gyration and the Quest for Functional Specialization

**DOI:** 10.3389/fnsys.2017.00096

**Published:** 2017-12-18

**Authors:** Lazaros C. Triarhou

**Affiliations:** Laboratory of Theoretical and Applied Neuroscience and Graduate Program in Neuroscience and Education, University of Macedonia, Thessalonica, Greece

**Keywords:** brain evolution, cerebral cortex, comparative neuroanatomy, cytoarchitectonics, ontophylogeny

## Soul in the rind

In the past, the distance that cognitively separates humans from other mammalian species was explained by our possessing a soul that animals did not have. Ramón y Cajal (1933) reduced the difference between human and animal intelligence to the abundance and complexity of cortical association fibers, the number of which increases in proportion to the quantity of gray matter: “The large cerebra of the elephant, whale, ox, horse, etc., possess many projection cells but relatively scarce association cells.”

Today, the explanation for human intellect is that we possess a bigger telencephalon: the richness of mental life depends on the surface area of an expanded cerebral cortex, considered to be the seat of consciousness (de Duve, 2002).

## Creaseless to crinkled

The vertebrate cerebral cortex varies from the trilaminar reptilian to the hexalaminar mammalian form (Shepherd, 2011). The evolution from lissencephaly to gyrencephaly provided mammals with a means to accommodate more cerebral cortex within the confines of their cranial vault. The degree of cortical folding depends on the cortical surface, thickness, volume, and convolutedness (Hofman, 1985). An increase in the *gyrification index* (GI) correlates with the increase in brain mass in mammalian orders, including primates, cetaceans, carnivores, and ungulates (Pillay and Manger, 2007). Cetaceans are the most gyrencephalic mammals, regardless of brain mass, which is a finding explained by their post-terrestrial return to a marine environment (Manger et al., 2012).

The areal expansion and the gyration of the cortical surface commence prenatally in the developing human. Gyrated cortices feature multipotent basal radial glial cells that reside in the outer subventricular zone (Ghosh and Jessberger, 2013). During late developmental stages, asymmetrical cell divisions in the ventricular zone generate radial glial cells and intermediate progenitor cells; subsequently, the latter divide symmetrically in the subventricular zone to produce multiple types of neurons (Rakic, 1988). The evolution of this two-step pattern of neurogenesis is theorized to have played an important role in the amplification of cell numbers underlying the radial and tangential cortical expansion (Martínez-Cerdeño et al., 2006). Cytoskeletal rearrangements also appear to be crucial for the development of gyrated brains (Nielsen et al., 2010).

The folding of the cerebral cortex has been attributed to a relative increase in the expansion of the superficial layers relative to deep layers and to the dissipation of the in-plane mechanical forces generated by the tangential cortical surface expansion (Van Essen, 1997; Bayer and Altman, 2006; Mota and Herculano-Houzel, 2015; Ronan and Fletcher, 2015). Axonal growth and synapse formation happen along with gyrification, such that laminar and regional cytoarchitecture is intimately linked to cortical “connectomics” and thalamocortical projections (Karten, 2015).

The developmental and evolutionary mechanisms of cerebrocortical gyrification, and their malformations, have been investigated by means of neuroimaging and molecular genetic methods (Rash and Rakic, 2014). Certain genes involved in the process of cortical gyrification demonstrate altered transcriptional activity during the time-frame when convolutions appear (Nielsen et al., 2010).

The fact that there is a similar pattern in the gyration across members within a species, but a different pattern among species, indicates that cortical convolution is a genetically-programmed process (Nielsen et al., 2010). A technical way to address the question of gyrification is by genomic analyses before and after the appearance of gyration in diverse species; that is, by comparing the differential expression of identified genes between the lissencephalic embryonic stage and the primary-folded gyrencephalic stage, as Nielsen et al. (2010) did in the pig.

The DNA-associated protein Trnp1 regulates cortical expansion tangentially and radially. In mice, high levels of Trnp1 lead to cortical gyrification in an animal that is normally lissencephalic (Stahl et al., 2013). Another gene, *ARHGAP11B*, which is unique to humans, has been shown to promote basal progenitor cell generation in the subventricular zone and induce cerebrocortical gyrification after insertion into the mouse genome (Florio et al., 2015).

The *GPR56* gene encodes a heterotrimeric G-binding protein-coupled receptor expressed in cortical progenitor cells and required for normal cortical development; the GPR56 protein functions in cell adhesion and guidance (Nielsen et al., 2010; Rash and Rakic, 2014). A 15-base pair deletion in the regulatory region of *GPR56* was discovered in patients with familial seizures, mental disability, and bilateral cortical abnormalities in the frontal lobe around the Sylvian fissure, including Broca's area (Bae et al., 2014); in mice, *GPR56* overexpression led to an increase of cortical progenitor proliferation and influenced gyral patterning.

## Functional attributes

The issue of functional localization in the human cerebral cortex has journeyed from neuroanatomical phenomenology to hypothesis-driven neuropsychology and back. Classical neuroanatomists considered brain structure and function as one; they studied morphology from a histophysiological perspective, not as a mere parcellation of neurons (Jakob, 1939). Cajal, Brodmann, Economo, Koskinas, and the Vogts worked on the premise that morphological diversity reflected functional specifications, leaving it to future physiological and clinical studies to attribute functional individualities to anatomical subdivisions (Bartels and Zeki, 2005; Jones, 2008). Koskinas (1931) put it succinctly: “As a general principle, each physiological function presupposes a corresponding anatomical basis. From the precise knowledge of the structure of the cerebral cortex we may expect to shed light on issues of the utmost importance, such as the relationship between mental attributes and brain structure.” Examples of cytoarchitectonic subdivisions that reflect functional differentiation were found in motor, somatosensory, and visual fields in the frontal, parietal, and occipital lobes.

Cortical cytoarchitecture and myeloarchitecture are inextricable from neuronal connections (van den Heuvel et al., 2015). In defining cortical areas, connectivity is key; the guiding principle of neuroanatomists that cortical areas form parts of connectional networks is now being adopted by the neuroimaging community, including the streams of intrinsic cortico-cortical connections, the re-entrant projections from the thalamus, and their ontogeny (Jones, 2008).

The traditional hypothesis-driven paradigm faces new challenges (Frackowiak and Markram, 2015). The “piece-meal” research style we are used to cannot offer a full understanding of brain function; instead, an integrated, multilevel explanation seems imperative, comprising all organizational aspects of the nervous system, from DNA to behavior, and the cooperation of such different levels with each other.

Advances in neuroimaging have led to new knowledge about brain organization at a systems level, “a macro-scale road map for understanding perception, action, and cognition” (Badre et al., 2015). Neuroimaging researchers, equipped with ever more powerful magnets, seem content with locating a gyrus that corresponds to a Brodmann numbered area and referring a particular behavioral action to that location (Jones, 2008). They revived the notion of “cognitive brain mapping” and purport to unveil physical representations of cognition in living cerebral tissue; the term “activation” (adjustable pseudocolors generated by software) became an ambiguous catchall term (Smith, 2010). Typically, an activation occurs when two experimental conditions produce statistically-significant differences in relative, normalized signal strength between two neighboring anatomical regions. In fMRI, mental functions are taken as localized in cortical sites (“functional segregation”), a notion reflecting the old view that conscious processes must have a seat in the brain, rather than connections.

There are further limitations in decoding the results of functional magnetic resonance imaging (fMRI) (Haynes, 2015). Information contained in single voxels or voxel ensembles cannot be directly correlated with information encoded in single neurons because the method relies on the magnetization level of blood as an indirect marker of activity in pools of thousands of neurons in a nonlinear hemodynamic response. Signals can appear in disparate brain areas, not connected anatomically, and with different signal-to-noise ratios. Moreover, a positive signal in a fMRI can overestimate the information available at the neuronal level, influenced by the pattern of blood vessel drainage; conversely, absence of information in the fMRI does not mean absence of information at the level of local neuron populations (Haynes, 2015).

An inherent limitation of fMRI is resolution (macro-level). If we consider that the key to any behavioral outcome is the activity at the synapse (micro-level), we realize that today's imaging cannot reveal the ultimate morphological or chemical happenings that lead to behavior. There is a strong argument that it is not merely helpful to understand the nano-scale organization of the brain for insight into its function; it is a requisite (Südhof, 2017); a molecular understanding of the brain further necessitates taking into account the incessant neural plasticity, the non-synaptic communication between neurons, and the role of glia.

Cortical functions are integrative. Their underlying network commonality transcends parcellation and connectivity, especially with the thalamus, and is therefore crucial in defining any cortical area. Even white matter imaging methods, such as diffusion-tensor and diffusion-spectrum imaging, do not reveal the synaptic terminations of axons in the gray matter of the cerebral hemispheres (Jones, 2008).

Nonetheless, imaging techniques make it possible to study human representational space noninvasively in unprecedented ways, provided they are interpreted cautiously. Sizeable experimental data have been gathered in the effort to link particular behaviors to specific anatomical loci in the human cerebral hemispheres. A function is attributed to a cerebral lobe, cortical gyrus, lobule, or cytoarchitectonic area (Vandenberghe et al., 2001; Zysset et al., 2003; Rivera et al., 2005; Kitada et al., 2009; Sestieri et al., 2010). Brain imaging studies have gone as far as associating individualist, conservative and radical political ideologies to cortical areas—medial prefrontal cortex and temporoparietal junction, dorsolateral prefrontal cortex, and ventral striatum and posterior cingulate cortex, respectively (Zamboni et al., 2009)—and associating cortical gyri with economic-political decisions or voting behavior (Xia et al., 2015).

## The discrepancy of species

Pioneers of neuroanatomy, including Obersteiner, Flatau, Edinger, Retzius, Jakob, Ariëns-Kappers, Herrick, and Welker, placed emphasis on comparative neurology in their quest to understand the human brain in the context of growth, form, and function (Obersteiner, 1890; Retzius, 1896; Edinger, 1899; Flatau and Jacobsohn, 1899; Jakob and Onelli, 1913; Hofman and Johnson, 2011).

**Figure 1:**
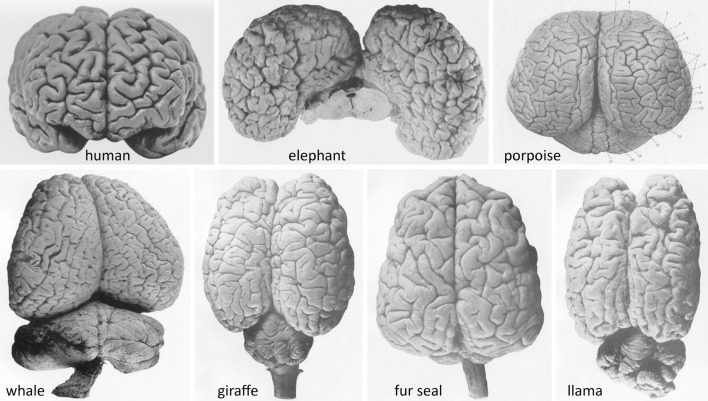
Brains of advanced gyrencephalic species depicted in classical neuroanatomical works (varying scales). **(Upper)**: Adult human *(Homo sapiens sapiens)* (Retzius, 1896, plate 57/1); African elephant *(Loxodonta africana)* (Jakob and Onelli, 1913, plate 38/262); and common porpoise *(Phocoena phocoena)* (Flatau and Jacobsohn, 1899, plate 6/1). **(Lower)**: Southern right whale *(Eubalaena australis)*; giraffe *(Giraffa camelopardalis)*; South American fur seal *(Arctocephalus australis)*; and llama *(Lama glama)* (Jakob and Onelli, plates 37/254, 30/206, 36/242, 27/176, respectively). Modern photographs of brains of over 175 species are available online (NeuroscienceLibrary.org) at the National Museum of Health and Medicine of the Armed Forces Institute of Pathology, Washington, D.C., which houses several renowned neuroanatomical collections, including the University of Wisconsin–Madison Comparative Mammalian Brain Collection (Fobbs and Johnson, 2011).

The tendency to ascribe a function to each lobule or gyrus of the human cerebral hemispheres comes in sharp contrast to the fact that, for the plethora of mammals with rich gyration patterns of the cerebral cortex (Figure 1), we have very little data that allow us to attribute specific functions to each anatomically-defined ensemble. What do all these gyri and cytoarchitectonic areas do? It would be a paradox to concur that natural selection produced human gyri for specific functional outcomes, while in other species the presence of numerous gyri just serves to fill the cranial cavity.

The twentieth century in neuroscience has centered around a small number of models, such as mice, rats, cats, dogs, rabbits, and monkeys (so-called “classic laboratory animals”; Nielsen et al., 2010). The trend accelerated as genomic sequences and molecular genetic tools became available for specific species, leading to a “bottleneck.” It is now realized that the comparative study of species from different phyletic lineages can be useful for the formulation and critical testing of hypotheses (Brenowitz and Zakon, 2015). Evidently, mammalian species that have not yet been studied outnumber those studied. In an attempt to explain anatomical structure in tandem with functional specialization in complex brains, neuroscience research of the twenty-first century should establish a coherent denominator by extending research to a range of richly-gyrated, “exotic” animals that have not been studied extensively or at all. There are unique brain collections in comparative anatomy museums worldwide that remain unexploited (Iwaniuk, 2010). An ambitious plan is to generate a phylogenetic tree of functional cortical cartography; in other words, a comparative neurology that takes into account the fourth dimension—the macro-time scale of evolution (Triarhou, 2008). Only then can the blueprint of cortical gyration be understood in conjunction with its microstructure and integrative functional output.

Along that line, substantial progress is noted in the study of brain structure in gyrencephalic species beyond primates, such as the dromedary (Simon, 1965), llama (Welker et al., 1976), horse (Cozzi et al., 2014), hippopotamus (Butti et al., 2014), rhinoceros (Manger, 2011; Bhagwandin et al., 2017), elephant seal, and sea lion (Sawyer et al., 2016; Turner et al., 2017), not to mention the extensive literature on proboscidea (Dexler, 1907; Jakob, 1909; Janssen and Stephan, 1956; Haug, 1966; Cozzi et al., 2001; Shoshani et al., 2006; Jacobs et al., 2011; Herculano-Houzel et al., 2014) and cetacea (Tower, 1954; Haug, 1970; Walløe et al., 2010; Butti et al., 2011; Mortensen et al., 2014).

Common anatomical landmarks were documented across gyrencephalic species early in the scientific history of this topic (Flatau and Jacobsohn, 1899; Jakob and Onelli, 1913). The question emerges whether there are homologous areas, circuits, and pathways that mediate perceptual, visual, auditory, somatosensory, and other processes or, reversely, whether there are homologous behaviors, conceivably subserved by different cytoarchitectonic areas. Or is the assumption of homologies among species increasingly difficult to sustain, as Frackowiak and Markram (2015) reasoned? Beyond the basics, what is the morphofunctional nature of gyri? Which cytoarchitectonic areas are conserved in larger gyrated brains and which diverge? What is the layer distribution of neuronal types and how are they assembled into neocortical circuits? Histology, histochemistry, microelectrode recordings and biochemical analyses, which historically yielded landmark discoveries in neuroscience, should not be abandoned or underestimated as techniques for the future.

Computational methods of mapping and quantifying cortical area layout, such as the original comparative approach of Chaplin et al. (2013) in simian primates, are also meaningful in the quest to probe form and function in larger gyrated brains.

In the accounts of phylogenetic evolution, the occasional bias in the resolution of cladogram branches in favor of *Homo sapiens* was pinpointed (Sandvik, 2009). Perhaps the answers to the problems outlined above would help us to gain a broader understanding of neocortical gyration, and a less anthropocentric interpretation of neurobiology.

## Author contributions

The author confirms being the sole contributor of this work and approved it for publication.

### Conflict of interest statement

The author declares that the research was conducted in the absence of any commercial or financial relationships that could be construed as a potential conflict of interest.
